# Decoding hypnotic consciousness: neural and experiential insights into induced and ideomotor suggestions

**DOI:** 10.1093/nc/niag019

**Published:** 2026-05-18

**Authors:** Juliette Gélébart, Alexandre Fouré, Romain Quentin, Ursula Debarnot

**Affiliations:** University Lyon 1, Inter-University Laboratory of Human Movement Biology (LIBM), EA 7424, 27–29 boulevard du 11 Novembre 1918, 69622, Villeurbanne, France; University Lyon 1, Inter-University Laboratory of Human Movement Biology (LIBM), EA 7424, 27–29 boulevard du 11 Novembre 1918, 69622, Villeurbanne, France; EDUWELL Team, Lyon Neuroscience Research Center (CRNL), CNRS (UMR 5292), INSERM (U1028), University Lyon 1, 95 boulevard Pinel, 69500 Bron, France; University Lyon 1, Inter-University Laboratory of Human Movement Biology (LIBM), EA 7424, 27–29 boulevard du 11 Novembre 1918, 69622, Villeurbanne, France; Institut Universitaire de France, Paris, France

**Keywords:** hypnosis, induction, ideomotor suggestion, neurophenomenology, functional connectivity, consciousness

## Abstract

Hypnotic induction and ideomotor suggestions provide a powerful framework for investigating the remarkable capacity of verbal influence to reshape conscious experience, cognition, and motor control. We employed a multimodal, neurophenomenological approach combining high-density electroencephalography, cardiorespiratory, and behavioral monitoring, as well as first-person reports across three conditions: a resting-state baseline, a hypnotic induction, and an ideomotor challenge involving either a suggested arm rigidity with attempted movement or a voluntary wakeful simulation used as a behavioral control condition. Electroencephalography (EEG) analyses revealed that hypnosis induction-related changes unfolded gradually, beginning with parieto-occipital alpha suppression and increased theta activity, followed by enhanced frontoparietal theta connectivity and reduced parasympathetic cardiac modulation. These results confirm and extend prior findings, showing that hypnotic induction suggestions involve an active, high-arousal, top-down reorganization of large-scale brain networks. During ideomotor challenge, participants displayed distinct behavioral patterns, classified as tremblers and non-tremblers, despite reporting comparable disruptions in agency. Phenomenological reports clarified these differences: tremblers attempted movement despite experiencing the action as involuntary or constrained, while non-tremblers refrained from acting due to a felt impossibility or an inhibited motor command. EEG connectivity in tremblers specifically showed increased frontoparietal gamma and reduced delta connectivity, consistent with enhanced sensorimotor prediction error signaling under motor conflict, relative to voluntary simulation. Together, these findings demonstrate that hypnosis dynamically reconfigures neural connectivity and subjective experience depending on suggestion type. They further support predictive coding and dissociation-based accounts of agency disruption and underscore the value of neurophysiological and neurophenomenological methods for advancing consciousness science and informing clinical applications.

Highlights

- Neurophenomenology links electroencephalography connectivity, physiology, behavior, and experience
- Hypnotic induction involves fronto-parietal network reorganization without relaxation
- Ideomotor suggestions reveal a dissociation between agency experience and motor output
- Fronto-parietal gamma and delta connectivity shifts reflect motor prediction conflict
- Predictive processing explains altered agency and conscious control under hypnosis

## Introduction

Hypnosis is increasingly understood as a top-down regulatory process where verbal suggestions can elicit mental representations that dynamically influence perceptual, cognitive, and ideomotor processes, leading to measurable changes in subjective experience and behavior (Oakley and Halligan 2009, Terhune et al. 2017). Hypnosis sessions typically unfold in a sequence of partially overlapping functional phases, shaped by distinct classes of verbal suggestions (Elkins et al. 2015): induction suggestions that promote attentional capture, absorption, and expectancy (Zahedi et al. 2024a); a central phase involving specific suggestions such as ideomotor ones, which modulate motor control and the sense of agency, a key component of intentionality (Martin and Pacherie 2019); and a de-induction phase in which suggestions reorient attention and restore ordinary perception and behavior. Underlying these phases, the top-down regulation exerted by hypnotic suggestions reflects a multifaceted interaction between attentional control, executive monitoring, and expectancy-related processes, all shaped by sociocultural and psychological factors (Lynn et al. 2015, Landry et al. 2017). This complexity gives rise to inter-individual variability in hypnotic responsiveness (Raz 2011, Terhune et al. 2017), as empirical evidence indicates that individuals may engage diverse cognitive strategies in response to similar suggestions, which complicates the isolation of suggestion-specific responsiveness across subjective, behavioral, and neural levels (Galea et al. 2010, Oakley and Halligan 2013). Consistent with this view, prior work has shown that hypnotic depth and responsiveness are not linearly related to induction duration, and that individuals may enter or fail to enter a hypnotic state at different points during the induction phase (Woody et al. 2005, Zahedi and Sommer 2022). To date, and despite advances in the cognitive neuroscience of hypnosis, the neurophysiological and phenomenological mechanisms underlying induction and ideomotor suggestions, the most commonly used in hypnosis, remain largely elusive (Landry et al. 2017, Terhune et al. 2017). Yet, understanding these mechanisms could offer insights into broader theories of consciousness dynamics and agency regulation, while informing the development of hypnosis-based clinical interventions (Jensen et al. 2017, Timmermann et al. 2023).

Advancing the understanding of induction and ideomotor suggestions requires addressing methodological limitations that contribute to variability in findings and compromise replicability (Landry et al. 2017, Terhune et al. 2017, Martin and Pacherie 2019). These discrepancies primarily arise from experimental designs lacking rigorous comparisons between hypnotic states with matched awake control conditions, thereby hindering the dissociation of induction-related effects from those driven by suggestion content, expectancy, or task demands (Braffman and Kirsch 1999, Connors et al. 2012, Terhune and Cardeña 2016, Jensen et al. 2017). More recent work has further emphasized that hypnotic induction is often confounded with suggestion delivery and contextual expectancy, calling for designs that explicitly disentangle these components (Kekecs et al. 2024). Although findings remain mixed, a broad consensus suggests that hypnosis engages frontal networks linked to top-down cognitive control, most consistently evidenced by increased theta-band spectral power (4–7 Hz) following induction (Jensen et al. 2015, Landry et al. 2017). However, accumulating electroencephalography (EEG) evidence indicates that hypnotic responsiveness is not reducible to changes in oscillatory power within a single frequency band (de Pascalis 2024). Instead, hypnosis appears to modulate large-scale neural connectivity across multiple frequency ranges, from delta (1–4 Hz) to gamma (>30 Hz), highlighting functional integration and network reconfiguration as key mechanisms (Jensen et al. 2015, de Pascalis 2024). Accordingly, studies using interregional coherence measures, such as the imaginary component of coherency (iCOH; Nolte et al. 2004a), have linked distinct connectivity changes to hypnosis induction (Egner et al. 2005). For instance, Jamieson and Burgess (2014) reported that highly suggestible individuals show reduced beta connectivity in fronto-central and occipital regions, along with increased theta connectivity in a central-parietal hub, whereas Terhune et al. (2011) found that this hub exhibited reduced connectivity in the alpha band. More recently, Niedernhuber et al. (2024) reported reduced interhemispheric frontoparietal connectivity in hypnosis-experienced individuals, facilitating information transfer between the left parietal and right frontal regions, independently of the self-reported hypnotic depth. These findings may suggest that hypnosis induction suggestions promote cortical information flow along a frontal-to-posterior hierarchy and enhances frontoparietal integration, supporting attentional modulation and immersive hypnotic experiences. However, given the heterogeneity of experimental designs and control conditions in the field of hypnosis, replication and multimodal approaches remain essential to clarify the specificity and functional significance of these connectivity patterns.

Variability in mental strategies when responding to specific hypnotic suggestions further poses a significant empirical issue, as similar behavioral outcomes may arise from different underlying cognitive and neural processes (Derbyshire et al. 2004, Cardeña 2014, Lynn et al. 2015). This issue is exemplified in challenge-ideomotor paradigms, where participants are suggested to imagine their arm becoming stiff like a bar of iron, and then attempt to bend it, creating a conflict between actual somatosensory feedback (the arm can bend) and the suggested percept of rigidity (a bar of iron cannot bend) (Winkel et al. 2006, Galea et al. 2010). Previous studies using this paradigm have revealed two motor response profiles: some individuals show visible arm trembling, while others remain still, despite both reporting comparable experiential responsiveness and alteration in the sense of agency (Winkel et al. 2006, Galea et al. 2010). This dissociation indicates that subjective compliance with the suggestion does not uniquely determine motor output, and that different strategies may be deployed to resolve the imposed conflict. Addressing this variability requires neurophenomenological approaches that integrate first-person subjective experiences (Northoff and Ventura 2025), providing a relevant framework for understanding the neural mechanisms underlying ideomotor phenomena (Petitmengin and Lachaux 2013, Timmermann et al. 2023). To date, neuroimaging studies of hypnosis have focused on direct ideomotor suggestions of arm-paralysis, in which participants are instructed not to move a limb, typically without being challenged to attempt movement, and compared them to voluntary simulated motor inhibition (Cojan et al. 2009, Cardeña et al. 2013, Deeley et al. 2013a, 2013b). In these paradigms, suggested paralysis has been associated with increased prefrontal activity linked to executive control, enhanced precuneus involvement, and reduced motor–premotor connectivity, consistent with a mechanism emphasizing self-monitoring and altered agency rather than direct motor inhibition (Ward et al. 2003, Cojan et al. 2009, Deeley et al. 2013a). Importantly, in such direct ideomotor suggestions, imagined and actual motor states are congruent, requiring no neural adaptation. By contrast, challenge suggestions involve a conflict between imagined rigidity and actual motor capacity, and resolving this mismatch demands heightened attention and neural adaptation, making the top-down process more cognitively demanding (Zahedi et al. 2024a). Given growing evidence that hypnotic effects are primarily reflected in changes in large-scale connectivity rather than local activation, EEG investigations using challenge-based ideomotor paradigms are particularly well suited to identify distinct cognitive–motor strategies and to map their neural implementation within a well-characterized behavioral and phenomenological response framework.

Overall, the neurophysiological and subjective mechanisms underlying the top-down regulation that governs responsiveness to induction and ideomotor suggestions remain poorly understood. In the present study, we pursued two complementary objectives. First, we examined the neurophysiological dynamics associated with induction-related suggestions, compared to a resting-state control condition (Ctrl). Second, we investigated the neurophenomenological mechanisms underlying hypnotically suggested arm rigidity within a challenge-based ideomotor paradigm, relative to a simulated voluntary condition performed while awake. To address these questions, we employed a within-subject, counterbalanced controlled design and a multimodal assessment framework combining neural, physiological, behavioral, and first-person subjective measures.

## Material and method

### Participants

The study protocol was approved by the Research Ethic committee of the University of Lyon (2023-10-19-002) and followed the principles expressed in the Declaration of Helsinki. Consent forms for participation in initial hypnotizability screening and experimental session were provided. Subjects were excluded if they reported any history of neurological or psychiatry disease, or took medication or drugs. We screened 40 healthy right-handed individuals using the Harvard Group Scale of Hypnotic Susceptibility: FormA [4]. This test involved a structured hypnotic induction followed by a series of 12 suggestions targeting sensory, motor, and volitional alterations. Only participants who achieved a minimum score of 6 out of 12 items, including successful compliance with the challenged ideomotor suggestion, were included in the experimental study. Our final sample was composed by 23 healthy right-handed (11 women, mean age of 24.75 ± 3.78) with medium to high level of hypnotizability (8.80 ± 1.82). Three participants were excluded from the analyses as the ideomotor challenge suggestion failed during the experiment, leading them to bend their arm during the Rigid trials.

### Experimental design

**Figure 1:**
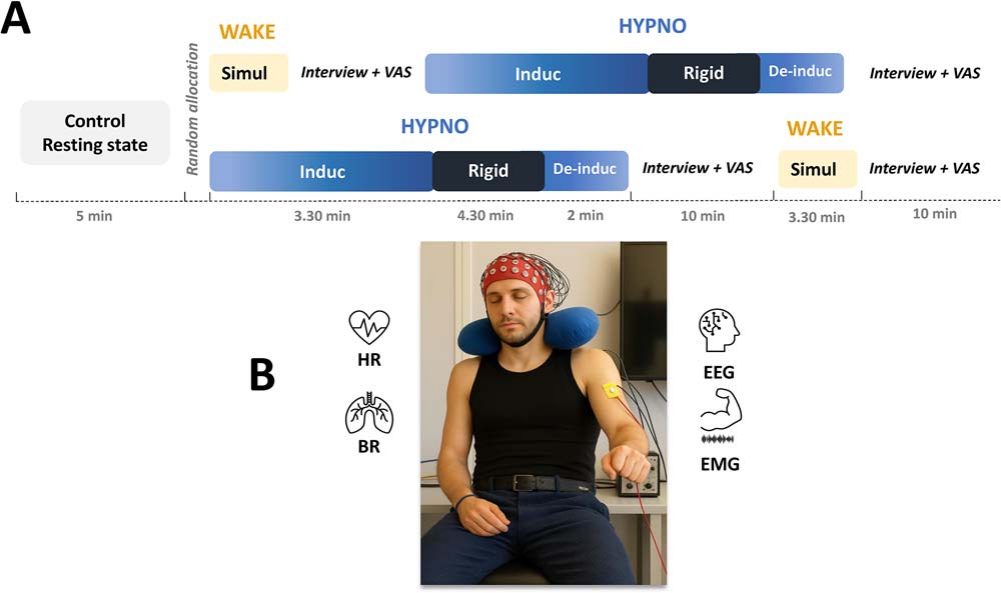
Experimental design and multimodal neurophysiological assessments. (A) Following resting-state (Ctrl), participants were randomly assigned to two counterbalanced experimental conditions, with the WAKE session involving the awake simulation of impossible arm-bending trials (Simul), and the HYPNO session including neutral induction-type suggestions and then challenged-ideomotor suggestions targeting the left arm Rigid trials. Each session concluded with a first-person phenomenological interview and VAS to collect subjective experiences. (B) Neurophysiological and multimodal assessments included continuous recordings of EEG, HR, BR, and EMG activity of the biceps, and triceps muscles. Note: the face shown has been replaced with an AI-generated image for anonymization purposes.

The experiment was conducted in a quiet, controlled environment to minimize external disturbances. One experimenter (J.G.) was responsible for ensuring the real-time acquisition and monitoring of multimodal assessments, while a second experimenter (U.D.), a trained hypnotist, administered the hypnotic instructions and guided the participants throughout the experimental session including first person interview. This division of roles ensured the seamless integration of data collection and the consistent delivery of hypnotic protocols. The participant was seated in an armchair, a cushion placed around the neck, with continuous recordings of heart rate and breathing rate (HR/BR), left-arm electromyography (EMG), and 64-channel EEG throughout the session. The experiment began with a 5-min eyes-closed resting state, during which participants were instructed to remain still and avoid focused thoughts, providing a baseline for neurophysiological measures, including EEG and HR/BR. Participants then proceeded with the waking ordinary state of consciousness condition (WAKE), followed by the hypnotic condition (HYPNO), with the order of conditions counterbalanced across participants (Fig. 1).

During the WAKE condition, participants were instructed to simulate the inability to bend their left forearm over the upper arm, acting “as if they were trying to bend their left arm but were unable to do so,” while emphasizing that they could “feel free to perform the simulation in their own manner”. Then, participants were asked to raise their left arm, stretched out in front of them, with their fist and eyes closed. Each simulated trial (Simul) began with a verbal “go,” lasted ~4 s (±1 s), and was followed by a 5-s break during which subject maintained the position. A total of 10 trials were conducted, and condition lasted 3.28 ± 0.49 min.

The HYPNO condition began with induction suggestions based on Gruzelier’s model (Gruzelier 1998), starting with visual fixation. Participants were instructed to extend their left arm forward and focus their gaze on their thumb until the arm naturally lowered, their eyelids gradually closed, and their hand rested on their thigh. On average, the duration of attentional induction was 1.30 ± 1.02 min. Then, the relaxation and multimodal imagery induction-type suggestions were used to promote progressive absorption and attentional disengagement from the external environment beginning with “You can focus on your exhalation. . . Every time you exhale, pay attention to how your muscles relax. . . Continue to let this sensation of relaxation spread. . ..” This phase was followed by a standardized counting sequence, similar to that used in the Harvard test, inviting the participant to move into an increasingly absorbed and internally oriented experiential state with each number beginning from 20. During this phase, the suggestions aimed to increase the participant’s internal focus absorption, and the dissociation of consciousness between the physical and the mental like: “You will move into a state of comfort in which you will be able to perform movements I will ask you to make. . .Pay only attention to my voice. . .In this state of deep relaxation, you may find yourself feeling both present and elsewhere. . . both focused and distracted . . . neither tense nor relaxed. . ..” On average, the induction phase lasted 3.23 ± 0.24 min.

Then levitation suggestions were provided for the left arm and hand, and once the desired position in front of the participant was achieved, the experimenter began ideomotor suggestions, indicating that their left arm was becoming so rigid, like a “metal bar” that it could no longer be bent. They were then invited to attempt bending the “metal bar,” which was suggested as impossible, for 4 s (± 1 s) during 10 trials (Rigid), and rigidity suggestions were maintained across the 5-s inter-trials to keep the adherence of the subject. For instance: “It [subject’s arm] is becoming stiff … more and more stiff … rigid …like a bar of iron … and you know how difficult … how impossible it is to bend a bar of iron like your arm… Try to bend it [4 s]… you can stop trying”. The termination of the hypnosis followed a standardized procedure, including a suggestion to shift focus back to the physical environment and restore typical expectations regarding the influence of one’s actions and perception of external stimuli. This phase systematically included behavioral verification by the experimenter (e.g., voluntary movements of all fingers, hand, and the arm previously in catalepsy), followed by an exchange with the participant. The duration of challenged ideo-motor condition was 4.36 ± 0.57 min.

Before each condition, participants rated their current state of alertness using the Stanford Sleepiness Scale (Hoddes et al. 1972). A minimum score of 3 (i.e., relaxed, awake, not at full alertness, responsive) was required before starting the next condition. After both the WAKE and HYPNO conditions, a structured debriefing session was conducted to gather first-person phenomenological data on participants’ subjective experiences and motor control strategies. This involved targeted questions such as “What/how do you do when you hear ‘go’?” or “What/how do you do when you hear ‘try to bend the bar’?”, depending on the condition just completed. The interview was conducted by U.D., who is trained in phenomenological interviewing techniques aimed at guiding participants to explicit their subjective experiences, focusing on the action (mental/motor) and intentionality (self-agency) (Petitmengin 2006), which lasted ~5 min. They were further asked to rate on a visual analog scale (VAS) their left arm muscular fatigue, and their subjective strength deployed during trials. Participants were also asked to rate the subjective depth of their hypnotic experience following the HYPNO condition, using a 0–10 scale, where 0 indicated “not hypnotized at all” and 10 corresponded to “as deeply hypnotized as you have ever been”. The time between the two conditions was 9 min 4 s ± 0.52 s. Finally, to confirm the effectiveness of the arm rigidity suggestion, participants completed the Catalepsy Questionnaire (Hagenaars et al. 2006) assessing their perception of both arms during the ideo-motor suggestions. The closer the score was to 10, the more effective the arm rigidity suggestion was in the affected arm. Two isometric maximum voluntary contractions of the triceps and biceps brachii were performed, with 2 min of recovery between each, to allow the normalization of the EMG data for analysis.

### E‌EG recordings and preprocessing

EEG was recorded from 64 EEG electrodes (ActiCAP snap, BrainProducts®, Munich, Germany) localized according to the 10–20 system on participants’ heads. The impedance of each electrode was kept below 10 kOhm. The initial sampling rate was 1000 Hz, and data were down-sampled off-line to 256 Hz. Reference was on mastoids (TP9 and TP10) and converted to average reference offline. The EEG signal was amplified by BrainAmp amplifiers and recorded with the BrainVision Recorder software (Brain Products®, Munich, Germany). The EEG data were processed and analyzed offline using the BrainVision® System (BrainProducts®, Munich, Germany). A 1 Hz high-pass filter and a 50 Hz notch filter were applied to the EEG signal. EEG recordings for each condition were segmented into 3-s epochs beginning 500 ms post-movement onset to capture stable neural activity associated with sustained motor effort while minimizing transient artifacts related to task initiation. Independent component analysis was used to remove eye movements and muscles artifacts. Any segments containing artifacts over 250 μV were discarded from the analysis. Fast Fourier Transforms (FFT) and phase-coherence analysis were performed on the artifact-cleaned epochs of the Ctrl, Mid-Induc, End-Induc, Rigid, and Simul conditions.

#### Spectral analysis

Each 3-s EEG segment was windowed with a Hanning tapering window prior to computing the power spectra using the FFT in five spectral band: δ (0.5–3.5), θ (4–7.5 Hz), α (8–12 Hz), β (13-30 Hz), and γ (31–80 Hz) and computed for each electrode. The power content, expressed as μV^2^ for each condition, was determined as the average power across the 3-s segments of the EEG. For analysis, the relative power was calculated by dividing the sum of spectral power from all bins for each frequency bin within 0 and 80 Hz and each electrode.

#### Functional connectivity analysis

Functional connectivity was studied using EEG imaginary phase-coherence analysis between six pairs of regions (Frontal left: mean of FP1, AF3, AF7, F3, F5, F7; Frontal right: mean of FP2, AF4, AF8, F4, F6, F8; Central left: mean of FC3, FC5, FT7, C3, C5, T7, CP3, CP5, TP7; Central right: mean of FC5, FT7, C3, C5, T7, CP3, CP5, TP7; Parietal left: mean of P3, P5, P7, PO3, PO7; Parietal right: mean of P4, P6, P8, PO4, PO8) for all conditions (Ctrl, Mid-Induc, End-Induc, Rigid, and Simul) and for each frequency bands (δ, θ, α, β, and γ). Coherence is defined as the square of the cross-spectrum between electrodes, divided by the product of the power spectra of the individual electrodes (Nolte et al. 2004b). This calculation provides a measure of the consistency of the phase relationship between two signals, with values ranging from 0 to 1. However, the measure of coherence is very sensitive to volume conduction, which can lead to erroneous correlation estimates. To address this issue, we focused on iCOH, which has been proposed as an effective method for assessing brain connectivity based on the frequencies of brain signals (Nolte et al. 2004b). The iCOH is obtained by taking the imaginary part of the cross-spectrum of the electrodes and dividing it by the square root of the product of the individual electrodes’ power spectra. Thus, after performing the FFT of the signal for each spectral band power, the absolute value of the imaginary result was computed across the 3-s segments of the EEG. The total iCOH was calculated separately for each frequency band.

### Heart rate variability and breathing rate measures

The Hexoskin Biometric Shirt (Carré Technologies Inc., Montreal, Québec, Canada) was used with the Hexoskin Smart Device (datalogger) to measure the participants’ average HR and BR. This wearable monitor is a compression shirt with built-in cardiac and breathing sensors sit around the thorax and abdomen. The cardiac sensors are analog ECG recording at 256 Hz and the respiratory sensors are dual channel respiratory inductance plethysmography sensors recording at 128 Hz. HR was detected from the ECG and recorded every second in units of beats per minute. The BR was recorded every second in units of breaths per minutes (bpm). The datalogger collects data from the sensors and stores it internally. Markers were added at the beginning and end of each state. At the end of the experimental session, the data were extracted from the datalogger to a computer “via” USB. HR analysis, including artifact detection and extraction of time and frequency domains, was performed using MATLAB software (R2019a®). In the time domain, the root means square successive difference (RMSSD, in ms) was calculated. In the frequency domain, the power in the low (LF: 0.04–0.15 Hz) and high (HF: 0.15–0.40 Hz) frequency bands were quantified, and the LF/HF ratio was subsequently calculated. Additionally, exploratory cardiorespiratory coupling was assessed by computing cross-correlations between the HR and BR time series. For each condition (Ctrl, Mid-Induc, End-Induc, Simul, Rigid), the maximum correlation coefficient was extracted as an index of coupling strength, used here as a global, exploratory marker of cardio-respiratory interactions.

### Surface electromyography

Five surface EMG electrodes (Asept foam adhesive electrodes, medico electrodes international LTD) were attached to the subject’s left arm over the biceps brachii and long head of the triceps brachii in accordance with Surface Electromyography for the Non Invasive Assessment of Muscles guidelines (Hermens et al. 2000). The reference electrode was attached to the wrist bone. Prior to the application of the electrodes, the areas around the electrode attachment sites were shaved and rubbed so that skin impedance could be greatly reduced. A cotton pad soaked with alcohol was then used to clean the skin. The electrodes were placed parallel to the muscle fibers with inter-electrode distance of 2 cm. EMG signal was acquired using data acquisition software LabChart 8 and sampled at a frequency of 2 kHz. Then, the EMG signal was filtered by a 50 Hz notch filter to remove line noise and a 20–500 Hz fourth-order band-pass filter. From the EMG signal of the Rigid and Simul conditions, the root mean square (RMS) was calculated over a 3-s period for each trial. A Maximum Voluntary Contraction-normalization was performed to allow comparison of RMS values between subjects. Thus, obtained RMS values represent the percentage of maximum force exerted by each participant during two isometric voluntary contraction per muscle. Average over all subjects (*n* = 20) was computed for the Rigid and Simul conditions. Additionally, spectral analysis of the EMG signal was performed in the 20–120 Hz frequency range during the Simul and Rigid conditions, for both biceps and triceps muscles. Power spectral density was computed using Welch’s method over the same 3-s period for each trial, and mean power values across the frequency band were calculated for subsequent statistical analysis.

### Phenomenological analysis

To analyze participants’ experiential reports from the explicitation interviews conducted after the Rigid and Simul trials, we used an emergent coding scheme following a grounded theory approach (Glaser 1992). The goal of the interviews was to probe both “what” participants experienced during the trials and “how” they mentally and physically enacted the suggested tasks, with a specific focus on motor strategies and intentionality. Two raters, one of whom was blind to participants’ experience, analyzed the full transcript of all reports. Each rater initially reviewed the data independently and developed a list of non-overlapping experiential dimensions that could classify all responses with the minimal number of distinct categories. Dimensions were then compared and discussed, focusing specifically on motor engagement and intention during the trials. Inter-rater agreement was particularly strong for motor-related experiential dimensions, which were consistently and independently identified by both raters. Minor discrepancies on other categories were resolved through discussion.

### Statistical analyses

Using Python (version 3.10) in Visual Studio Code (Microsoft, 2022), differences in the power spectrum across all electrodes were statistically assessed using a cluster-based permutation test comparing conditions pairwise. The t-statistic was calculated for each cluster, which was defined as two or more spatially contiguous electrodes where the t-statistics of power spectrum exceeded a chosen threshold of alpha level of *P* < .05 and α_cluster_ = 0.01. A total of 1000 permutations were performed to establish a null distribution of the test statistic. The subsequent statistical analyses were conducted using JASP®software (version 0.16.1.0). To test for connectivity differences, Repeated-measures analysis of variance (ANOVArm) were first conducted on iCOH values between the Ctrl, Mid-Induc, End-Induc, and then between Simul and Rigid, across pairs of regions (Fr, Fl, Cr, Cl, Pr, Pl) for each frequency band separately. For each set of comparisons, a False Discovery Rate correction (FDR) was applied across the five frequency bands to control for multiple comparisons (Benjamini and Hochberg 1995). Two-way ANOVArm, with conditions (Simul vs Rigid) as within factor and muscles (Biceps, Triceps) as between factor, was conducted on the RMS values from the EMG. To assess the difference in the coefficient of variation according to the trembling behavior, Mann–Whitney tests were computed for the Simul and Rigid conditions, for both the biceps and triceps brachii. To further examine whether condition-related effects were also reflected in the frequency domain, the same two-way repeated-measures ANOVA structure (conditions × muscles) was applied to mean EMG spectral power in the 20–120 Hz range. Exploratory analyses including trembling behavior as an additional between-subject factor were conducted separately (see supplementary file). To examine the effect of Ctrl and Induc conditions on one hand, and the Ctrl, Simul, and Rigid conditions on the other hand, on heart rate variability (RMSSD and LF/HF), and on BR, ANOVArm were performed. ANOVArm was also conducted on cardiorespiratory coupling across all five conditions (Ctrl, Mid-Induc, End-Induc, Simul, Rigid).

For all statistical analyses, corrections for multiple comparisons were applied where appropriate. For each ANOVAs, the Greenhouse–Geisser correction was applied when necessary. *Post hoc* analyses with Bonferroni correction for multiple comparisons were performed when significant effects or interactions were found following ANOVAs.

## Results

### Induction suggestions: theta-coupled fronto-parietal network reorganization tracks hypnotic depth and vagal disengagement

**Figure 2:**
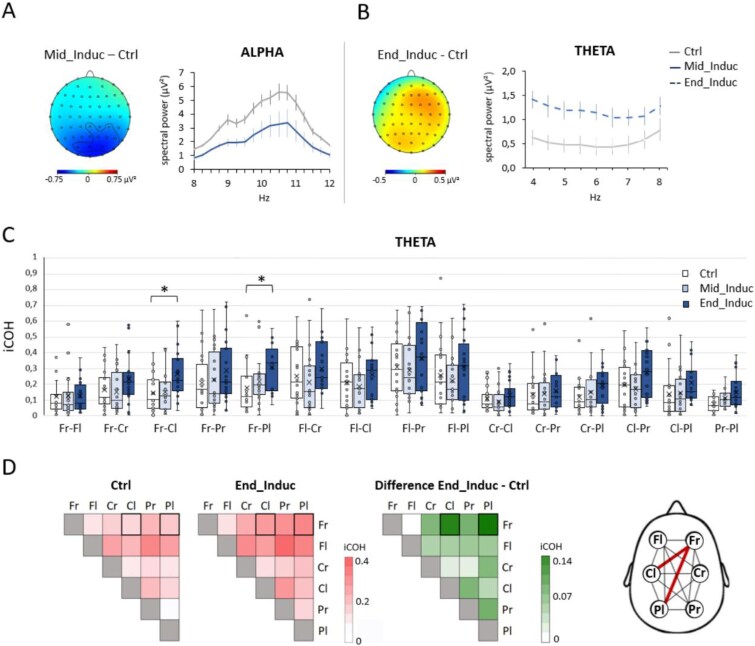
Spectral power and iCOH changes between resting state and hypnosis induction. (A) Spectral power analysis over electrodes belonging to significant clusters, illustrating both topographic and graphical differences in alpha power between the Mid_Induc and Ctrl conditions, and (B) theta power between the End_Induc and Ctrl conditions. (C) Theta-band iCOH changes across Ctrl, Mid_Induc, and End_Induc conditions for each brain region pair, with boxplots showing iCOH distribution, with the cross indicating the mean and the line the median. (D) Heatmaps display iCOH values for Ctrl and End_Induc conditions on a red gradient, while the green map highlights differences between the two conditions, with significant changes in bold. Brain connectivity map illustrates the increase in connectivity in the theta band, during the End_Induc compared to Ctrl condition state between right frontal region (Fr) and left central and parietal regions (Cl and Pl respectively). Fl: right frontal; Cr: right central; Pr: right parietal regions. *P < .05.

EEG pairwise spectral comparisons between the Ctrl and Induc conditions, using a cluster-based permutation test, revealed a significant decrease in alpha power during the Mid-Induc phase relative to Ctrl, predominantly over parieto-occipital electrodes (PO3, POz, PO4, PO8, P6, P4, P2, CPz, and CP4; 1 < *t*-values < 5, *P* = .041; Fig. 2A). Then, a generalized theta power enhancement was observed during the End-Induc condition across all electrodes (25 < *t*-values < 175, *P* = .001; Fig. 2B).

To test for connectivity differences, iCOH values were compared between the Ctrl, Mid-Induc, and End-Induc conditions, across pairs of regions (left and right frontal: Fr, Fl, central: Cr, Cl, and parietal: Pr, Pl regions). In the theta frequency band, the iCOH revealed a main effect of conditions (*F*_(2,38)_ = 4.145, *P_FDR* = 0.024, η^2^_p_ = 0.179), of regions (*F*_(14,266)_ = 11.822, *P_FDR* < 0.001, η^2^_p_ = 0.384), and a conditions*regions interaction (*F*_(28,532)_ = 1.507, *P_FDR* = 0.031, η^2^_p_ = 0.050; Fig. 2C). *Post hoc* comparisons revealed that, during the End-induc condition, the right frontal region exhibited significant greater connectivity with the left parietal (*P* = .016, d = 0.631) and left central regions (*P* = .034, d = 0.570) in the theta band, compared to Ctrl (Fig. 2D). For the delta, alpha, beta, and gamma frequency bands, only a main effect of the regions was observed (δ: *F*_(14,266)_ = 8.412, *P_FDR* < 0.001, η^2^_p_ = 0.280; α: *F*_(14,266)_ = 10.243, *P_FDR* < 0.001, η^2^_p_ = 0.353; β: *F*_(14,266)_ = 5.245, *P_FDR* < 0.001, η^2^_p_ = 0.231; γ: *F*_(14,266)_ = 4.200, *P_FDR* < 0.001, η^2^_p_ = 0.184).

**Figure 3:**
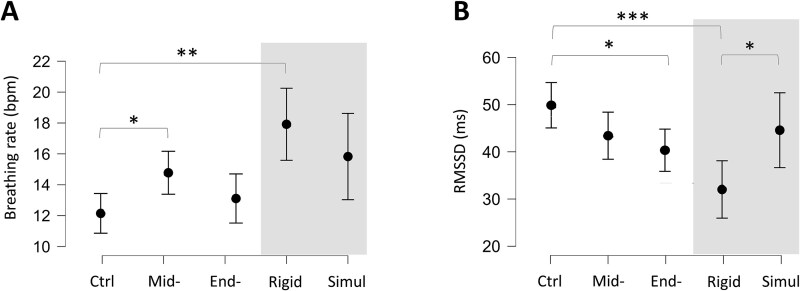
Physiological responses across different phases of HYPNO condition and WAKE simulation. (A) Breathing rate across conditions, with a higher rate during the Mid-Induc phase compared to Ctrl (*P < .05) and a global increase during Simulation (**P < .01). (B) RMSSD across conditions, showing a significant decrease during End-Induc compared to Ctrl (*P < .05), and significant differences between End-Induc, Rigid, and Simul (***P < .001, *P < .05). (C) Data are presented as means ± SEM.

Analysis of physiological BR across the induction revealed a main effect of condition (F_(1.879,31.939)_ = 3.862, *P* = 0.03, η^2^_p_ = 0.387), with a higher BR during the Mid-Induc condition compared to Ctrl (*P* = .03, d = 0.479). No differences were observed between Ctrl and End-Induc (*P* = .96, d = 0.052), or between Mid- and End-Induc conditions (*P* = .27, d = −0.011; Fig. 3A). Heart-rate variability analysis showed no effect of condition on the Low frequency/Hight frequency ratio (F_(2,21)_ = 0.76, *P* = .48, η^2^_p_ = 0.017). However, a main effect of the RMSSD (F_(2,28)_ = 4.80, *P* = .016, η^2^_p_ = 0.255), with *post hoc* analysis indicating lower RMSSD values during the End-Induc condition compared to Ctrl (*Post hoc* test, *P* = .015, d = −0.980), suggesting a reduction in parasympathetic activity across induction suggestions (Fig. 3B). Cardiorespiratory coupling analysis did not reveal any statistical difference across all conditions (F_(4,56)_ = 1.062, *P* = .384, η^2^_p_ = 0.071). Noteworthy, breathing rate analysis revealed a main effect of condition (F_(1.837,31.222)_ = 6.253, *P* = .006), with a higher breathing rate during Rigid compared to Ctrl (*P* = .005), accompanied by a RMSSD reduction in Rigid compared to Ctrl (F_(2.28)_ = 9.692, *P* < .001, d = −1.558) and compared to Simul (*P* = .035, d = −0.971), suggesting higher parasympathetic activity during Rigid (Supplementary Fig. S1).

### Challenged ideomotor suggestions: behavioral and phenomenology responsiveness

In the Rigid condition, 20 out of 23 participants successfully responded to the ideomotor suggestion of “not being able to bend the iron bar” across all 10 trials, with a subjective rating of cataleptic phenomenon of 9.2 ± 1.5 out of 10 for the left arm and 2.4 ± 0.7 for the right arm. The level of hypnotic depth in the HYPNO condition was 7.43 ± 1.18 out of 10. As expected, most were classified as “Tremblers” (*n* = 16), exhibiting muscle tremors during the Rigid trials, while a smaller subset, the “Non-Tremblers” (*n* = 4), showed no visible tremors. This online observation of trembling behavior was supported by *post hoc* surface EMG analysis and participants’ subjective reports regarding their motor strategies. First, RMS values, which quantify muscle activity intensity by measuring the overall amplitude of the EMG signal, were compared across Simul and Rigid trials. No main effects were found for muscle (*F*_(1,35)_ = 1.329, *P* = .257, η^2^_p_ = 0.174), condition (*F*_(1,35)_ = 0.847, *P* = .364, η^2^_p_ = 0.067), or their interaction (*F*_(1,35)_ = 2.238, *P* = .144, η^2^_p_ = 0.105). Consistently, EMG spectral power analyses also revealed no significant main effects of muscle (F_(1,28_) = 0.598, *P* = .446, η^2^_p_ = 0.021), condition (F_(1,28)_ = 1.430, *P* = .242, η^2^_p_ = 0.05), or their interaction (F_(1,28)_ = 1.292, *P* = .265, η^2^_p_ = 0.044; see Supplementary Fig. S2). To assess dynamic changes in muscle tone, a coefficient of variation of RMS values was computed for each participant. This analysis revealed greater RMS variability in “Tremblers” compared to “Non-Tremblers” for both the biceps (U = 5.05, *P* < .001, rrb = 0.420) and triceps brachii (*U* = 10.00, *P* = .049, rrb = 0.871). These results suggest that “Tremblers” alternated between contraction during trials and partial relaxation between trials, whereas “Non-Tremblers” maintained sustained muscle contraction throughout.

Further insights were obtained from a first-person interview conducted after both the HYPNO and WAKE conditions focused on mental strategies used during the Rigid and Simul trials. The *post hoc* analysis revealed two main findings, based on a coding scheme developed following a grounded theory approach (Glaser 1992), which identified recurring themes directly from participants’ first-person accounts: (i) the mental strategies employed during the Rigid and Simul conditions were inconsistent both within and across participants; and (ii) “Tremblers” and “Non-Tremblers” differed in their experiential reports during Rigid trials. Regarding the main thematic clusters, “Tremblers” frequently described attempting the movement despite perceiving it as involuntary or constrained, whereas “Non-Tremblers” typically reported not trying at all, either because it felt impossible from the outset or due to an inability to initiate the motor command. In the Simul condition, where participants were asked to simulate the same task in a wakeful, non-hypnotic context, all individuals reported trying to perform the movement voluntarily. Most described engaging in active blocking strategies, such as mentally halting the command at a specific joint (e.g. the elbow or wrist), while a few reported not to move without invoking an internal sense of inhibition.

**Figure 4:**
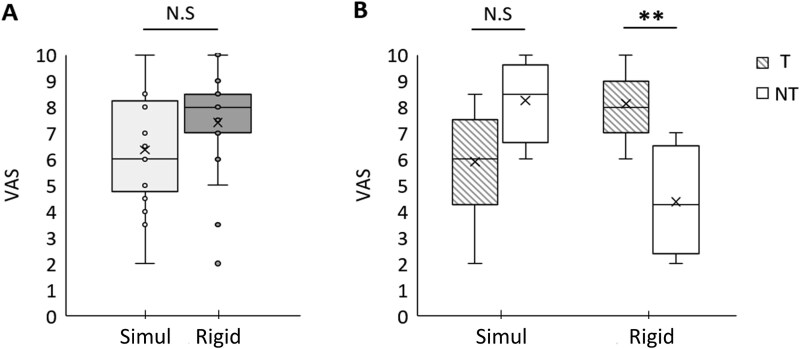
(A) Subjective strength deployed during Simul and Rigid trials assessed by each subject on a VAS. The central cross indicate the mean across subjects, and the line represents the median. (B) Mean subjective strength deployed for the “Tremblers” (T, *n* = 16) and “Non-Tremblers” (NT, *n* = 4) profiles. **P < .01.

These differences in mental strategies were also reflected in the subjective rating of perceived strength exertion (Fig. 4). A paired t-test conducted across all participants comparing the subjective ratings between the Rigid and Simul conditions revealed no significant difference (t = −0.947, *P* = .361, d = 0.253). However, when examining individual responses, it was observed that four participants, identified as “Non-Tremblers”, showed reversed ratings compared to the majority, reporting lower perceived strength exertion during Rigid compared to Simul.

### Challenged ideomotor suggestions reduced delta and enhanced gamma connectivity in right fronto-parietal networks

**Figure 5:**
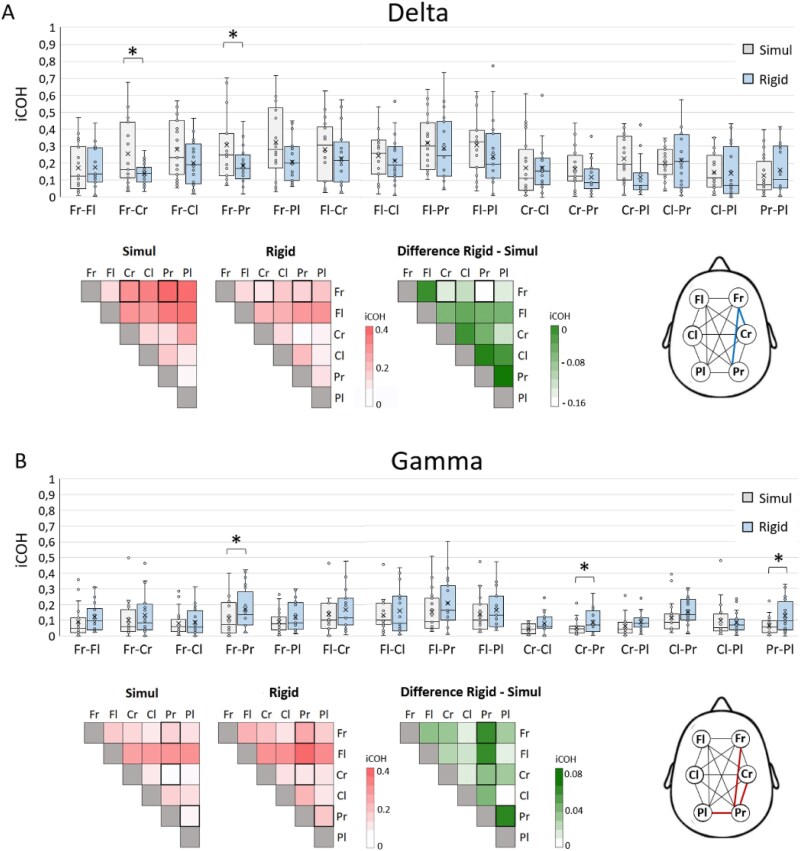
Differences in iCOH between simulated awake and hypnotic Rigid trials for delta and gamma frequency bands across brain regions. (A) Boxplots representing iCOH distributions across subjects, with the central cross indicating the mean and the line representing the median. Heatmaps display iCOH values for Simul and Rigid conditions on a red gradient, while the green map highlights differences between the two conditions, with significant changes in bold. Brain connectivity map illustrates lower delta band iCOH in Rigid compared to Simul (blue) between right frontal (Fr) and right central (Cr) and parietal (Pr) regions. (B) Similar representations for the gamma frequency band: Boxplots show iCOH distributions, with the mean and median indicated. Heatmaps illustrate iCOH for Simul and Rigid on a red gradient, with a green map representing the state differences and significant effects highlighted in bold. Brain connectivity map illustrates the increase in connectivity in the gamma band, during Rigid compared to Simul between right parietal region (Pr) and right central (Cr), right frontal (Fr) and left parietal (Pl) regions. *P < .05.

Given the differences in mental strategies used during Rigid trials, only “Tremblers” were included in the following EEG analyses (*n* = 16). Power spectrum differences between the Rigid and Simul conditions did not reveal any significant clusters for any frequency bands. Changes in iCOH in the delta and gamma band revealed a main effect of conditions (δ: *F*_(1,15)_ = 5.843, *P_FDR* = 0.03, η^2^_p_ = 0.320; γ: *F*  _(1,15)_ = 5.08, *P_FDR* = 0.04, η^2^_p_ = 0.253) and a main effect of regions (δ: *F*_(14,210)_ = 6.719, *P_FDR* < 0.001, η^2^_p_ = 0.239; γ: *F*_(14,210)_ = 3.26, *P_FDR* < 0.001, η^2^_p_ = 0.178), but no conditions *regions interaction (δ: *F*_(14,210)_ = 1.295, *P_FDR* = 0.21, η^2^_p_ = 0.097; γ: *F*_(14,210)_ = 1.163, *P_FDR* = 0.31, η^2^_p_ = 0.072). Results showed that during Rigid, the right frontal region exhibited lower connectivity with the right parietal (*P* = .023, d = −0.445) and central (*P* = .033, d = −0.714) regions in the delta band compared to Simul (Fig. 5A). Conversely, in the gamma band, the right parietal region showed increased connectivity with the right frontal (*P* = .036, d = 0.593), right central (*P =* .031, d = 0.641), and left parietal (*P* = .033, d = 0.587) regions during Rigid compared to Simul (Fig. 5B). For the theta, alpha, and beta frequency bands, only a main effect of regions was observed (θ: *F*_(14,210)_ = 3.67, *P_FDR* < 0.001, η^2^_p_ = 0.197; α: *F*_(14,210)_ = 8.33, *P_FDR* < 0.001, η^2^_p_ = 0.347; β: *F*_(14,210)_ = 5.28, *P_FDR* < 0.001, η^2^_p_ = 0.181).

## Discussion

This study investigated the neural and experiential dynamics elicited across hypnotic induction, and provides, the first neurophenomenological investigation of challenge-based ideomotor suggestions. Our findings show that the transition from a control resting state to hypnosis is characterized by progressive and state-dependent neurophysiological reconfigurations, consistent with active, top-down regulation modulation. Specifically, we observed an early induction-related changes marked by a reduction of parieto-occipital alpha power, followed by a generalized increase in theta power and strengthened theta-band functional connectivity between right frontal cortex and left parietal-central regions. During challenge-based ideomotor suggestions, participants classified as Tremblers exhibited a qualitative distinct pattern of large-scale cortical reorganization, characterized by increased gamma-band connectivity within a fronto-parietal network, and concomitant reductions in delta-ban connectivity between right frontal and parietal sites.

Regarding hypnotic induction, our data revealed significant changes from the control resting state to the Induc phase (i.e. Mid), marked by a decrease in alpha power over parieto-occipital regions. This early modulation is consistent with reduced processing of external stimuli and a reallocation of attentional resources toward internally oriented states (Panda et al. 2023, Niedernhuber et al. 2024). Rather than indexing hypnotic depth, it likely reflects the onset of attentional decoupling and perceptual reweighting, preparing the neural landscape for the suggestion-related processing and partial dissociation from the external environment (Jamieson and Burgess 2014). In this respect, the observed pattern shares neurophysiological similarities with the hypnagogic transition at sleep onset, where alpha suppression accompanies shifts in attentional control with partial loss of awareness (Tanaka et al. 1997, Wu et al. 2021). Notably, these neural changes occurred alongside increased in breathing rate and without modulation of cardiac vagal tone relative to the resting Ctrl condition. This dissociation indicates that hypnotic induction does not correspond to a passive relaxation response, but rather to a cognitively engaged state characterized by active attentional regulation in the absence of parasympathetic dominance, consistent with neurocognitive models emphasizing active top-down modulation during hypnosis (Terhune et al. 2017). Toward the end of induction, a broader transformation emerged, characterized by a robust increase in theta power across the scalp, a frequency consistently associated with sustained attention, mental imagery, and cognitive flexibility (de Pascalis 2024). This spectral change was accompanied by increased theta-band functional connectivity between right frontal and left parietal-central regions, echoing frontoparietal network configurations implicated in hypnotic absorption and altered self-related processing (Oakley and Halligan 2009, Fernandez et al. 2022, Niedernhuber et al. 2024). Such cross-hemispheric coupling suggests the recruitment of large-scale control networks supporting internal representation and executive monitoring, rather than mere sensory disengagement. Importantly, this transition was accompanied by reduced heart rate variability, reflecting decreased parasympathetic modulation rather than autonomic quiescence. This finding contrasts with the parasympathetic enhancement typically observed in meditation or relaxation-based hypnosis (Fernandez et al. 2022, de Benedittis 2024), indicating that hypnotic absorption is not contingent upon physiological relaxation. Instead, the co-occurrence of strengthened frontoparietal coordination and reduced vagal tone suggests a functional dissociation between cognitive engagement and autonomic regulation, possibly reflecting the sustained mental effort required to maintain immersive, suggestion-driven states. Taken together, these findings suggest that hypnotic induction is not a unitary relaxation response, but a staged brain–body transition, beginning with selective sensory decoupling and attentional reorientation and evolving toward large-scale network integration underlying absorption and executive monitoring, orchestrated by verbal suggestions. In this sense, hypnosis emerges as a distinct neurocognitive state, functionally autonomous from classic relaxation paradigms and grounded in dynamic reconfigurations of neural connectivity rather than global downregulation of arousal (Bauer et al. 2022).

Turning to ideomotor suggestions, our findings show that during the challenge-based rigidity condition, “Tremblers” and “Non-Tremblers” exhibited distinct motor responses, with the former showing rhythmic muscle oscillations both during and between trials, and the latter maintaining sustained muscular rigidity throughout. These patterns are consistent with previous studies reporting similar muscular distinctions, although those were limited to single-trial ideomotor suggestion challenges (Winkel et al. 2006, Galea et al. 2010). The present findings extend previous observations by revealing these motor distinctions across multiple trials and, through first-person interviews, suggest that they are associated with distinct mental strategies. Importantly, all participants were able to recall and describe their experience during the hypnotic Rigid trials, consistently reporting altered sense of agency, which was accompanied either by an effective motor attempt (“Tremblers”) or by the absence of any movement (“Non-Tremblers”). These findings are consistent with Deeley et al. (2013a), who showed preserved motor intention alongside involuntary motor inhibition during suggested limb paralysis. Similarly, in our study, awareness of the hypnotic experience and the subjective sense of involuntariness were preserved across participants. However, the dissociation between “Tremblers” and “Non-Tremblers” reveals a graded spectrum of motor expression rather than a binary presence or absence of agency. While both groups experienced diminished volitional control, “Non-Tremblers” were characterized by the absence of observable movement patterns, suggesting a distinct mode of motor implementation and agency attribution rather than a “deeper” loss of control. This divergence indicates that phenomenological access to agency can vary independently of conscious awareness, even under identical suggestions. Such variability underscores the importance of considering individual response strategies when interpreting hypnotic effects and reinforces the value of combining neural, behavioral, and first-person measures to capture the multifaceted nature of ideomotor phenomena.

Our main result revealed a reorganization of cortical connectivity in Tremblers, characterized by increased gamma-band coupling within right fronto-parietal and bilateral parietal regions, alongside a reduction in delta-band connectivity between right frontal and parietal sites. Previous oscillatory models of hypnosis have emphasized the role of cross-frequency interactions, notably theta–gamma coupling, as a mechanism through which slow oscillations modulate faster dynamics during hypnotic responding (Gruzelier 1998, Jamieson and Burgess 2014, Jensen et al. 2015). While these frameworks provide a useful descriptive background, the present findings are more coherently interpreted within a predictive processing account. Within predictive coding, gamma-band activity is considered a marker of unresolved prediction errors propagated across hierarchical levels, rather than motor commands or generic binding processes (Friston 2010, Banaie Boroujeni et al. 2021). Accordingly, enhanced gamma-band connectivity may reflect the neural integration of persistent prediction errors during challenged ideomotor suggestions, resulting from the coexistence of precise motor predictions and heightened sensory precision, a configuration known to disrupt the sense of agency (Martin and Pacherie 2019). This interpretation is consistent with predictive models of altered agency in hypnosis, in which suggestions modify high-level priors while lower-level sensory and motor signals remain active (Terhune et al. 2017, Martin and Pacherie 2019, Zahedi et al. 2024a). The concomitant reduction in delta-band connectivity may reflect a weakening of low-frequency integrative dynamics, allowing prediction errors to be maintained within higher-order fronto-parietal control networks (Morillon et al. 2019, de Pascalis 2024). Notably, these oscillatory changes were localized in right fronto-parietal regions contralateral to the suggested left arm, corresponding to areas repeatedly implicated in motor monitoring, inhibition, and altered experiences of agency during hypnosis and suggested paralysis (Cojan et al. 2009, Cojan et al. 2013, Deeley et al. 2013a, Niedernhuber et al. 2024).

From a conceptual viewpoint, our behavioral, subjective and autonomic findings jointly support recent predictive coding accounts of hypnotic ideomotor suggestion (Martin and Pacherie 2019, Zahedi et al. 2024b). Although both “Tremblers” and “Non-Tremblers” reported a loss in the sense of agency, the two profiles diverged markedly in their behavioral and phenomenological responses. “Tremblers” attempted to perform the suggested movement despite its perceived impossibility, producing rhythmic motor activity, while “Non-Tremblers” exhibited no overt movement and described a sense of motor command inhibition or blockage. From a predictive coding perspective, this dissociation reflects alternative strategies for minimizing sensorimotor prediction errors. “Tremblers” may engage in active inference, generating proprioceptive prediction errors through motor attempts in an effort to reconcile internal models with incoming sensory feedback (Kawato 1999). This pattern suggests reduced precision assigned to the suggestion-induced non-agency prior, permitting the propagation of unresolved sensorimotor prediction errors rather than their complete suppression at the motor level. In contrast, “Non-Tremblers” may rely more strongly on perceptual inference, assigning greater precision to the suggestion-based prior and attenuating the influence of conflicting sensory feedback. Together, these profiles exemplify the core mechanism of precision-weighting of prediction errors, whereby hierarchically organized sensorimotor and associative systems dynamically regulate the relative precision of ascending prediction errors and top-down expectations to preserve coherence in motor output and subjective experience Importantly, this dissociative configuration extends beyond motor behavior to autonomic regulation. In line with Hilgard’s dissociation model, bodily and sensory signals remain processed under hypnosis while becoming functionally decoupled from executive awareness (Hilgard 1992). Within a predictive processing framework, this may reflect altered precision weighting of prediction errors, allowing parasympathetic responses to persist without updating higher-order representations of conscious control or agency (Barrett and Simmons 2015, Seth and Friston 2016, Zahedi et al. 2024a).

Several limitations and perspectives should be acknowledged when interpreting the present findings. First, the experimental design does not fully dissociate the effects of hypnotic induction from those of suggestion and expectancy. A substantial body of work has shown that expectancy and contextual framing can account for a significant portion of hypnotic responding, sometimes independently of formal induction procedures, and that balanced placebo designs are better suited to isolate these components (Braffman and Kirsch 1999, Kekecs et al. 2024). Accordingly, induction-related effects in the present study should be interpreted cautiously and not as specific markers of induction alone. A second methodological limitation concerns the use of voluntary simulation as a comparison condition. Although simulated and hypnotically induced motor inhibition may appear behaviorally similar, prior EEG and neuroimaging studies indicate that they do not reliably engage the same cognitive or neural mechanisms (Ward et al. 2003, Cojan et al. 2009). More recent evidence further suggests that simulation relies on heterogeneous, strategy-dependent processes that diverge from the experiential and cortical signatures observed under hypnosis (Cojan et al. 2013, Franz et al. 2020). In this context, the WAKE–Simul condition should be considered a pragmatic behavioral benchmark controlling for voluntary inhibition and task demands, rather than a mechanistic proxy for hypnotic responding. Third, EEG analyses were restricted to “Tremblers”, and neurophysiological conclusions therefore apply specifically to this subgroup. The neural basis of tonic rigidity in “Non-Tremblers” remains unresolved; however, previous EEG and fMRI studies of hypnotic paralysis have shown preserved motor intention and monitoring despite absent movement, alongside altered motor network connectivity (Cojan et al. 2009, Cojan et al. 2013, Deeley et al. 2013a). Finally, while EEG metrics provide insights into large-scale functional dynamics associated with altered agency, the absence of source localization precludes precise anatomical inference, highlighting the need for future multimodal approaches. Beyond these methodological constraints, an important perspective emerging from the present findings concerns the broader functional scope of hypnosis-related motor modulation. Prior work has shown that hypnosis can not only inhibit movement but also facilitate motor performance through strength-enhancing suggestions, either during hypnosis or post-hypnotically (Takarada and Nozaki 2014, Nieft et al. 2024). Together, inhibitory and facilitative effects may therefore be viewed as complementary expressions of a common capacity of hypnosis to flexibly reconfigure motor control and agency-related processes.

To conclude, our findings reveal that hypnotic suggestions modulate brain–body dynamics through frequency-specific network reorganizations and divergent sensorimotor strategies. This supports predictive coding models of agency and underscores hypnosis as an active, non-unitary neurocognitive state. By integrating neurophysiological and phenomenological data, this study offers novel insights into how conscious experience is shaped by top-down processes. These results not only refine the theoretical understanding of hypnosis and volition, but also inform the development of individualized hypnotic interventions, particularly in contexts such as pain modulation, motor rehabilitation, and functional neurological disorders, where ideomotor suggestions may engage therapeutic reorganization of bodily awareness.

## Supplementary Material

niag019_Supplementary_materials

## Data Availability

The data underlying this article are available in https://osf.io/rjnbk/.

## References

[ref1] Banaie , Boroujeni K, Tiesinga P, Womelsdorf T. Interneuron-specific gamma synchronization indexes cue uncertainty and prediction errors in lateral prefrontal and anterior cingulate cortex. *elife* 2021;10:e69111. 10.7554/eLife.69111 34142661 PMC8248985

[ref2] Barrett LF, Simmons WK. Interoceptive predictions in the brain. *Nat Rev Neurosci* 2015;16:419–29. 10.1038/nrn3950 26016744 PMC4731102

[ref3] Bauer PR, Sabourdy C, Chatard B et al. Neural dynamics of mindfulness meditation and hypnosis explored with intracranial EEG: a feasibility study. *Neurosci Lett* 2022;766:136345. 10.1016/j.neulet.2021.136345 34785313

[ref4] de Benedittis G . Hypnotic modulation of autonomic nervous system (ANS) activity. *Brain Sci* 2024;14:249. 10.3390/brainsci14030249 PMC10968788 38539637

[ref5] Benjamini Y, Hochberg Y. Controlling the false discovery rate: a practical and powerful approach to multiple testing. *J R Stat Soc Ser B* 1995;57:289–300. 10.1111/j.2517-6161.1995.tb02031.x

[ref6] Braffman W, Kirsch I. Imaginative suggestibility and hypnotizability: an empirical analysis. *J Pers Soc Psychol* 1999;77:578–87. 10.1037/0022-3514.77.3.578 10510510

[ref7] Cardeña E . Hypnos and psyche: how hypnosis has contributed to the studyof consciousness. *Psychol Conscious Theory Res Pract* 2014;1:123–38.

[ref8] Cardeña E, Jönsson P, Terhune DB et al. The neurophenomenology of neutral hypnosis. *Cortex* 2013;49:375–85. 10.1016/j.cortex.2012.04.001 22579225

[ref9] Cojan Y, Waber L, Schwartz S et al. The brain under self-control: modulation of inhibitory and monitoring cortical networks during hypnotic paralysis. *Neuron* 2009;62:862–75. 10.1016/j.neuron.2009.05.021 19555654

[ref10] Cojan Y, Archimi A, Cheseaux N et al. Time-course of motor inhibition during hypnotic paralysis: EEG topographical and source analysis. *Cortex* 2013;49:423–36. 10.1016/j.cortex.2012.09.013 23211547

[ref11] Connors MH, Cox RE, Barnier AJ et al. Mirror agnosia and the mirrored-self misidentification delusion: a hypnotic analogue. *Cogn Neuropsychiatry* 2012;17:197–226. 10.1080/13546805.2011.582770 21899479

[ref12] Deeley Q, Oakley DA, Toone B et al. The functional anatomy of suggested limb paralysis. *Cortex* 2013a;49:411–22. 10.1016/j.cortex.2012.09.016 23351848

[ref13] Deeley Q, Walsh E, Oakley DA et al. Using hypnotic suggestion to model loss of control and awareness of movements: an exploratory FMRI study. *PLoS One* 2013b;8:e78324. 10.1371/journal.pone.0078324 24205198 PMC3804629

[ref14] Derbyshire SW, Whalley MG, Stenger VA et al. Cerebral activation during hypnotically induced and imagined pain. *NeuroImage* 2004;23:392–401. 10.1016/j.neuroimage.2004.04.033 15325387

[ref15] Egner T, Jamieson G, Gruzelier J. Hypnosis decouples cognitive control from conflict monitoring processes of the frontal lobe. *Neuroimage Clin* 2005;27:969–78. 10.1016/j.neuroimage.2005.05.002 15964211

[ref16] Elkins GR, Barabasz AF, Council JR et al. Advancing research and practice: the revised APA division 30 definition of hypnosis. *Int J Clin Exp Hypn* 2015;63:1–9. 10.1080/00207144.2014.961870 25365125

[ref17] Fernandez A, Urwicz L, Vuilleumier P et al. Impact of hypnosis on psychophysiological measures: a scoping literature review. *Am J Clin Hypn* 2022;64:36–52. 10.1080/00029157.2021.1873099 34748461

[ref18] Franz M, Schmidt B, Hecht H et al. Suggested deafness during hypnosis and simulation of hypnosis compared to a distraction and control condition: a study on subjective experience and cortical brain responses. *PLoS One* 2020;15:e0240832. 10.1371/journal.pone.0240832 33119665 PMC7595429

[ref19] Friston K . The free-energy principle: a unified brain theory? *Nat Rev Neurosci* 2010;11:127–38. 10.1038/nrn2787 20068583

[ref20] Galea V, Woody EZ, Szechtman H et al. Motion in response to the hypnotic suggestion of arm rigidity: a window on underlying mechanisms. *Int J Clin Exp Hypn* 2010;58:251–68. 10.1080/00207141003760561 20509067

[ref21] Glaser B . Basics of Grounded Theory Analysis. CA, Mill Valley: Sociology Press, 1992.

[ref22] Gruzelier J . A working model of the neurophysiology of hypnosis: a review of evidence. *Contemp Hypn* 1998;15:2–21.

[ref23] Hagenaars MA, Roelofs K, Hoogduin K et al. Motor and sensory dissociative phenomena associated with induced catalepsy: a brief communication. *Int J Clin Exp Hypn* 2006;54:234–44. 10.1080/00207140500528547 16581693

[ref24] Hermens HJ, Freriks B, Disselhorst-Klug C et al. Development of recommendations for SEMG sensors and sensor placement procedures. *J Electromyogr Kinesiol* 2000;10:361–74. 10.1016/S1050-6411(00)00027-4 11018445

[ref25] Hilgard ER . Divided consciousness and dissociation. *Conscious Cogn* 1992;1:16–31. 10.1016/1053-8100(92)90041-8

[ref26] Hoddes E, Dement WC, Zarcone V. The development and use of the Stanford sleepiness scale. *Psychophysiology* 1972;9:150.

[ref27] Jamieson GA, Burgess AP. Hypnotic induction is followed by state-like changes in the organization of EEG functional connectivity in the theta and beta frequency bands in high-hypnotically susceptible individuals. *Front Hum Neurosci* 2014;8:24. 10.3389/fnhum.2014.00528 25104928 PMC4109610

[ref28] Jensen MP, Adachi T, Hakimian S. Brain oscillations, hypnosis, and Hypnotizability. *Am J Clin Hypn* 2015;57:230–53. 10.1080/00029157.2014.976786 PMC4361031 25792761

[ref29] Jensen MP, Jamieson GA, Lutz A et al. New directions in hypnosis research: strategies for advancing the cognitive and clinical neuroscience of hypnosis. *Neurosci Conscious* 2017;3:nix004. 10.1093/nc/nix004 29034102 PMC5635845

[ref30] Kawato M . Internal models for motor control and trajectory planning. *Curr Opin Neurobiol* 1999;9:718–27. 10.1016/S0959-4388(99)00028-8 10607637

[ref31] Kekecs Z, Moss D, Whorwell PJ et al. Best practice recommendations for conducting and reporting controlled trials in clinical hypnosis research. *J Evid Based Integr Med* 2024;29:2515690X241274538. 10.1177/2515690X241274538 PMC11483803 39403729

[ref32] Landry M, Lifshitz M, Raz A. Brain correlates of hypnosis: a systematic review and meta-analytic exploration. *Neurosci Biobehav Rev* 2017;81:75–98. 10.1016/j.neubiorev.2017.02.020 28238944

[ref33] Lynn SJ, Laurence JR, Kirsch I. Hypnosis, suggestion, and suggestibility: an integrative model. *Am J Clin Hypn* 2015;57:314–29. 10.1080/00029157.2014.976783 25928681

[ref34] Martin JM, Pacherie E. Alterations of agency in hypnosis: a new predictive coding model. *Psychol Rev* 2019;126:133–52. 10.1037/rev0000134 30604989

[ref35] Morillon B, Arnal LH, Schroeder CE et al. Prominence of delta oscillatory rhythms in the motor cortex and their relevance for auditory and speech perception. *Neurosci Biobehav Rev* 2019;107:136–42. 10.1016/j.neubiorev.2019.09.012 31518638

[ref36] Niedernhuber M, Schroeder AC, Lercher C et al. An interhemispheric frontoparietal network supports hypnotic states. *Cortex* 2024;177:180–93. 10.1016/j.cortex.2024.05.008 38865762

[ref37] Nieft U, Schlütz M, Schmidt B. Increasing handgrip strength via post-hypnotic suggestions with lasting effects. *Sci Rep* 2024;14:23344. 10.1038/s41598-024-73117-0 39402088 PMC11473724

[ref38] Nolte G, Bai O, Wheaton L et al. Identifying true brain interaction from EEG data using the imaginary part of coherency. *Clin Neurophysiol* 2004;115:2292–307. 10.1016/j.clinph.2004.04.029 15351371

[ref39] Northoff G, Ventura B. Bridging the gap of brain and experience - converging Neurophenomenology with spatiotemporal neuroscience. *Neurosci Biobehav Rev* 2025;173:106139. 10.1016/j.neubiorev.2025.106139 40204159

[ref40] Oakley DA, Halligan PW. Hypnotic suggestion and cognitive neuroscience. *Trends Cogn Sci* 2009;13:264–70. 10.1016/j.tics.2009.03.004 19428287

[ref41] Oakley DA, Halligan PW. Hypnotic suggestion: Opportunities for cognitive neuroscience. *Nat Rev Neurosci* 2013;14:565–76. 10.1038/nrn3538 23860312

[ref42] Panda R, Vanhaudenhuyse A, Piarulli A et al. Altered brain connectivity and network topological organization in a non-ordinary state of consciousness induced by hypnosis. *J Cogn Neurosci* 2023;35:1394–409. 10.1162/jocn_a_02019 37315333

[ref43] de Pascalis V . EEG oscillatory activity concomitant with hypnosis and Hypnotizability. In: Linden JH, De Benedittis G, Sugarman LI, Varga K (eds.), The Routledge International Handbook of Clinical Hypnosis. 2024, 244–55.

[ref44] Petitmengin C . Describing one’s subjective experience in the second person: an interview method for the science of consciousness. *Phenomenol Cogn Sci* 2006;5:229–69. 10.1007/s11097-006-9022-2

[ref45] Petitmengin C, Lachaux JP. Microcognitive science: bridging experiential and neuronal microdynamics. *Front Hum Neurosci* 2013;7:617. 24098279 10.3389/fnhum.2013.00617 PMC3784800

[ref46] Raz A . Hypnosis: a twilight zone of the top-down variety few have never heard of hypnosis but most know little about the potential of this mind-body regulation technique for advancing science. *Trends Cogn Sci* 2011;15:555–7. 10.1016/j.tics.2011.10.002 22079011

[ref47] Seth AK, Friston KJ. Active interoceptive inference and the emotional brain. *Philos Trans R Soc Lond Ser B Biol Sci* 2016;371:20160007. 10.1098/rstb.2016.0007 PMC5062097 28080966

[ref48] Takarada Y, Nozaki D. Hypnotic suggestion alters the state of the motor cortex. *Neurosci Res* 2014;85:28–32. 10.1016/j.neures.2014.05.009 24973620

[ref49] Tanaka H, Hayashi M, Hori T. Topographical characteristics and principal component structure of the hypnagogic EEG. *Sleep* 1997;20:523–34. 10.1093/sleep/20.7.523 9322268

[ref50] Terhune DB, Cardeña E. Nuances and uncertainties regarding hypnotic inductions: toward a theoretically informed praxis. *Am J Clin Hypn* 2016;59:155–74. 10.1080/00029157.2016.1201454 27586045

[ref51] Terhune DB, Cardeña E, Lindgren M. Differential frontal-parietal phase synchrony during hypnosis as a function of hypnotic suggestibility. *Psychophysiology* 2011;48:1444–7. 10.1111/j.1469-8986.2011.01211.x 21496057

[ref52] Terhune DB, Cleeremans A, Raz A et al. Hypnosis and top-down regulation of consciousness. *Neurosci Biobehav Rev* 2017;81:59–74. 10.1016/j.neubiorev.2017.02.002 28174078

[ref53] Timmermann C, Bauer PR, Gosseries O et al. A neurophenomenological approach to non-ordinary states of consciousness: hypnosis, meditation, and psychedelics. *Trends Cogn Sci* 2023;27:139–59. 10.1016/j.tics.2022.11.006 36566091

[ref54] Ward NS, Oakley DA, Frackowiak RS et al. Differential brain activations during intentionally simulated and subjectively experienced paralysis. *Cogn Neuropsychiatry* 2003;8:295–312. 10.1080/13546800344000200 16571568

[ref55] Winkel JD, Younger JW, Tomcik N et al. Anatomy of a hypnotic response: self-report estimates, actual behavior, and physiological response to the hypnotic suggestion for arm rigidity. *Int J Clin Exp Hypn* 2006;54:186–205. 10.1080/00207140500528430 16581690

[ref56] Woody EZ, Barnier AJ, McConkey KM. Multiple Hypnotizabilities: differentiating the building blocks of hypnotic response. *Psychol Assess* 2005;17:200–11. 10.1037/1040-3590.17.2.200 16029107

[ref57] Wu J, Zhou Q, Li J et al. Decreased resting-state alpha-band activation and functional connectivity after sleep deprivation. *Sci Rep* 2021;11:484. 10.1038/s41598-020-79816-8 33436726 PMC7804319

[ref58] Zahedi A, Sommer W. Can hypnotic susceptibility be explained by bifactor models? Structural equation modeling of the Harvard group scale of hypnotic susceptibility - form a. *Conscious Cogn* 2022;99:103289. 10.1016/j.concog.2022.103289 35193060

[ref59] Zahedi A, Lynn S, Sommer W. How hypnotic suggestions work - a systematic review of prominent theories of hypnosis. *Conscious Cogn* 2024a;123:103730. 10.1016/j.concog.2024.103730 39032268

[ref60] Zahedi A, Lynn SJ, Sommer W. Cognitive simulation along with neural adaptation explain effects of suggestions: a novel theoretical framework. *Front Psychol* 2024b;15:1388347. 10.3389/fpsyg.2024.1388347 38966744 PMC11223671

