# Supplementary material for: Decoding hypnotic consciousness: neural and experiential insights into induced and ideomotor suggestions
Source: Neurosci Conscious. 2026 May 18;2026(1):niag019. doi: 10.1093/nc/niag019 (PMC13181418; doi:10.1093/nc/niag019)
Supplement: niag019_Supplementary_materials [file niag019_supplementary_materials.docx]

**Supplementary analysis**

Analysis of physiological BR across the induction revealed a main effect of condition (F_(1.879,31.939)_ = 3.862, *P* = 0.03, η²_p_ = 0.387), with a higher BR during the Mid-Induc condition compared to Ctrl (*P* = 0.03, d = 0.479). No differences were observed between Ctrl and End-Induc (*P* = 0.96, d = 0.052), or between Mid- and End-Induc conditions (*P* = 0.27, d = -0.011; Supp Fig.1). Heart-rate variability analysis showed no effect of condition on the Low frequency/Hight frequency ratio (F_(2,21)_ = 0.76, *P* = 0.48, η²_p_ = 0.017). However, a main effect of the Root Mean Square of Successive Differences (RMSSD; F_(2,28)_ = 4.80, *P* = 0.016, η²_p_ = 0.255), with post-hoc analysis indicating lower RMSSD values during the End-Induc condition compared to Ctrl (Post-hoc test, *P* = 0.015, d = -0.980), suggesting a reduction in parasympathetic activity during deepening of induction suggestions. Noteworthy, breathing rate analysis revealed a main effect of condition (F_(1.837,31.222)_ = 6.253, *P* = 0.006), with a higher breathing rate during Rigid compared to Ctrl (*P* = 0.005 ; Fig. 1), accompanied by a RMSSD reduction in Rigid compared to Ctrl (F_(2.28)_ = 9.692, *P* < 0.001, d = -1.558) and compared to Simul (*P* = 0.035, d = -0.971), suggesting higher parasympathetic activity during Rigid.


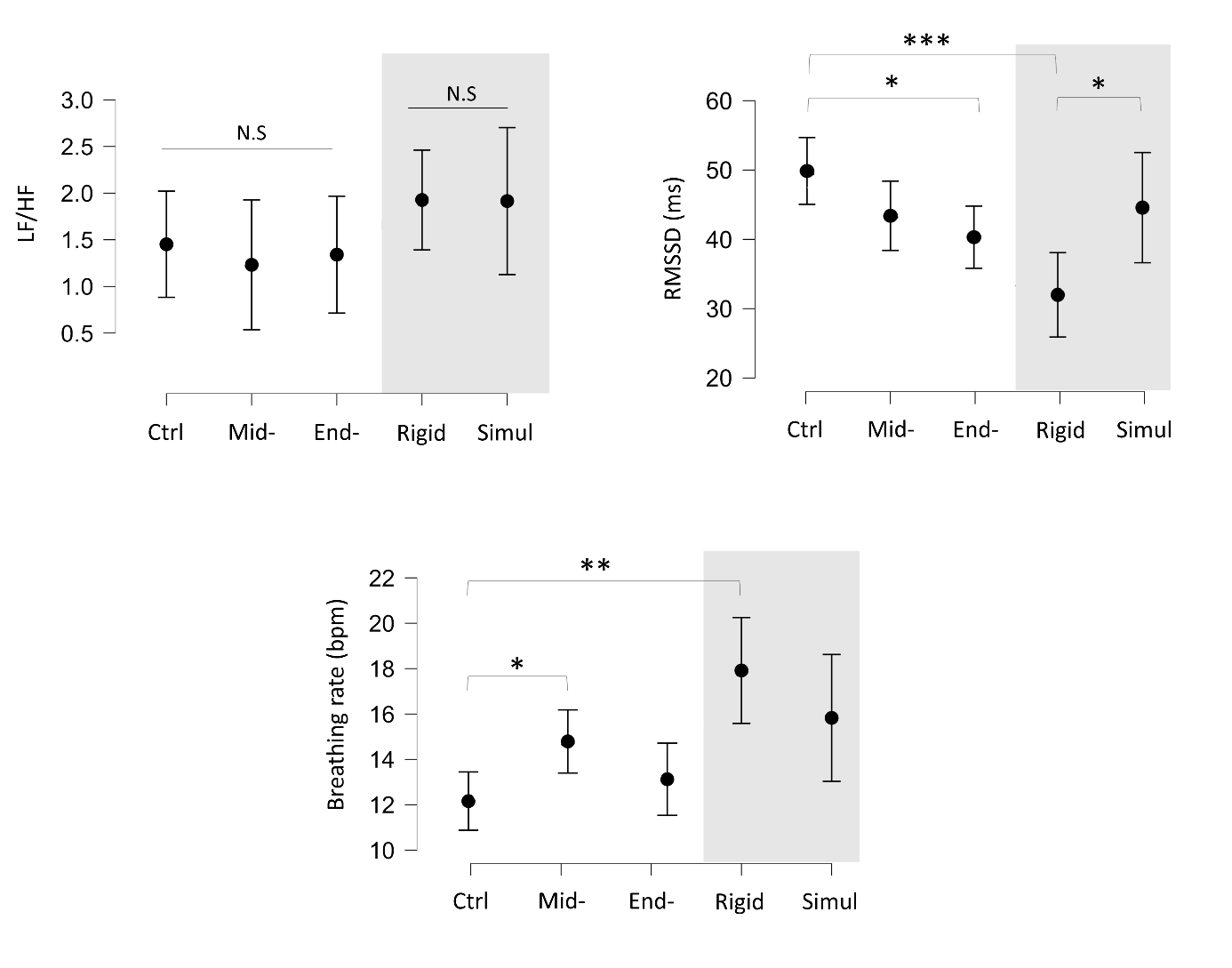


**Supp. Fig.1: Physiological responses across different phases of HYPNO condition and WAKE simulation**. A. Low frequency/High Frequency (LF/HF) ratio during Ctrl, Mid-Induc, End-Induc, Rigidity (Rigid), and Simulation (Simul) phases. B. Root Mean Square of Successive Differences values (RMSSD) across conditions, showing a significant decrease during End-Induc compared to Ctrl (* P < 0.05), and significant differences between End-Induc, Rigid, and Simul (*** P < 0.001, * P < 0.05). C. Breathing rate across conditions, with a higher rate during the Mid-Induc phase compared to Ctrl (*P < 0.05) and a global increase during Simulation (** P < 0.01). Data are presented as means ± SEM.

Exploratory EMG spectral power analyses conducted using repeated-measures ANOVA revealed no significant main effects of Condition (F_(1,28)_ = 0.347, p = 0.561, η²_p_ = 0.013), Muscle (F_(1,28)_ = 0.144, p = 0.708, η²_p_ = 0.005), or Trembling behavior (F_(1,28)_ = 1.139, p = 0.296, η²_p_ = 0.042), and no significant interactions (Condition x Muscle : F_(1,28)_ = 0.328, p = 0.572, η²_p_ = 0.012 ; Condition x Trembling behavior : F_(1,28)_ = 0.328, p = 0.572, η²_p_ = 0.012 ; Muscles x Trembling behavior : F_(1,28)_ = 0.149, p = 0.703, η²_p_ = 0.006 ; Condition x Muscles x Trembling behavior : F_(1,28)_ = 0.273, p = 0.606, η²_p_ = 0.010). Representative examples of EMG activity and time–frequency spectrograms illustrating trembling and non-trembling motor behavior are presented in Supp Fig.2.


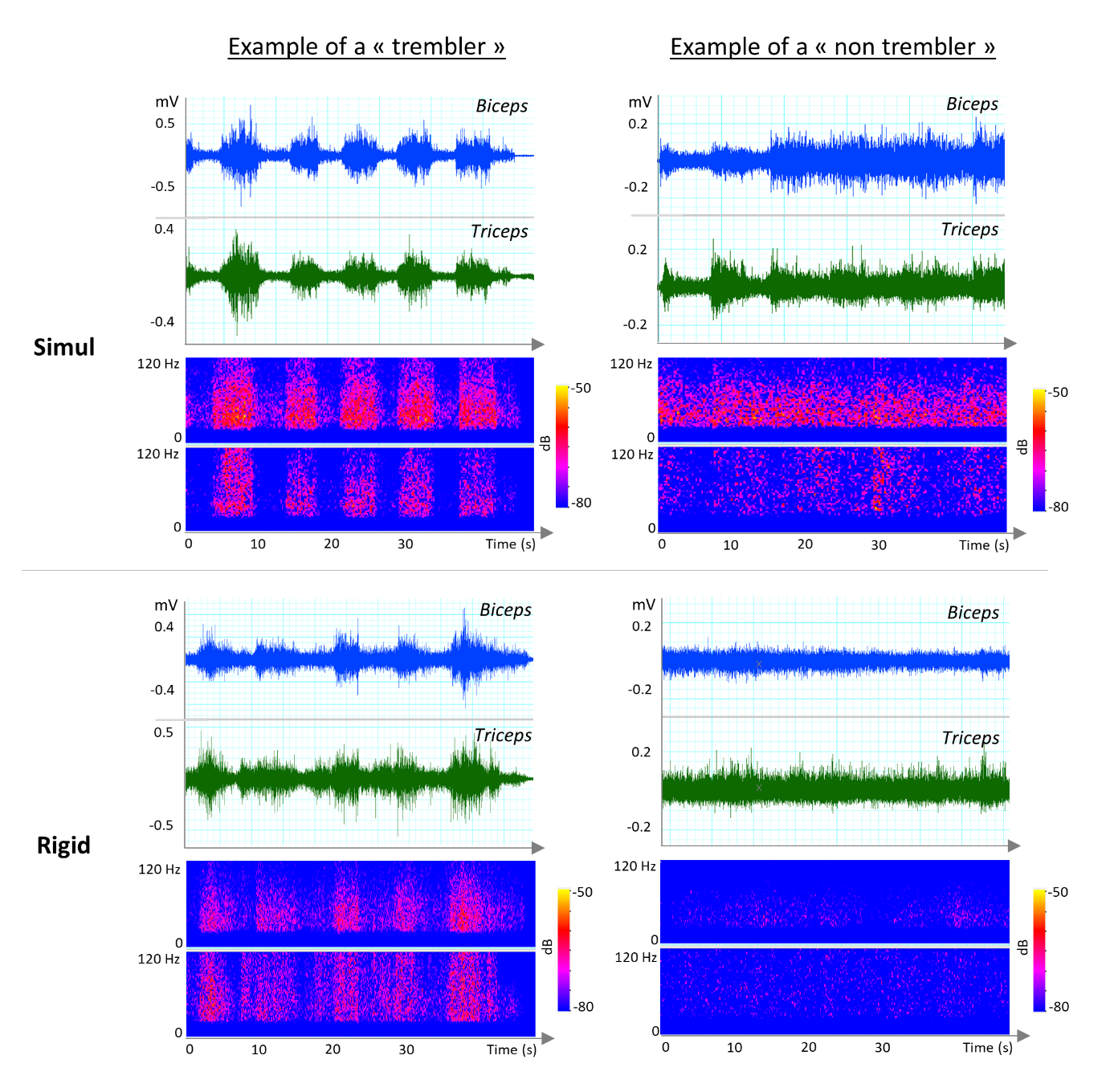


**Supp. Fig.2: Representative examples of EMG activity and time–frequency spectrograms illustrating trembling and non-trembling motor behavior.** Raw EMG signals from the biceps (blue) and triceps (green) muscles and their corresponding spectrograms are shown for a representative Trembler participant (left column) and a Non-Trembler participant (right column), independently of experimental condition (Simul or Rigid). These examples highlight qualitatively distinct activation patterns, characterized by intermittent, burst-like activity in Tremblers and more sustained, tonic activation in Non-Tremblers.
